# Point Mutations Associated with Organophosphate and Carbamate Resistance in Chinese Strains of *Culex pipiens quinquefasciatus* (Diptera: Culicidae)

**DOI:** 10.1371/journal.pone.0095260

**Published:** 2014-05-01

**Authors:** Minghui Zhao, Yande Dong, Xin Ran, Zhiming Wu, Xiaoxia Guo, Yingmei Zhang, Dan Xing, Ting Yan, Gang Wang, Xiaojuan Zhu, Hengduan Zhang, Chunxiao Li, Tongyan Zhao

**Affiliations:** 1 Beijing Institute of Microbiology and Epidemiology, State Key Laboratory of Pathogens and Biosecurity, Department of Vector Biology and Control, Beijing, China; 2 Anhui Medical University, Hefei, China; Weizmann Institute of Science, Israel

## Abstract

Acetylcholinesterase resistance has been well documented in many insects, including several mosquito species. We tested the resistance of five wild, Chinese strains of the mosquito *Culex pipiens quinquefasciatus* to two kinds of pesticides, dichlorvos and propoxur. An acetylcholinesterase gene (ace1) was cloned and sequenced from a pooled sample of mosquitoes from these five strains and the amino acids of five positions were found to vary (V185M, G247S, A328S, A391T, and T682A). Analysis of the correlation between mutation frequencies and resistance levels (LC_50_) suggests that two point mutations, G247S (r^2^ = 0.732, P = 0.065) and A328S (r^2^ = 0.891, P = 0.016), are associated with resistance to propoxur but not to dichlorvos. Although the V185M mutation was not associated with either dichlorvos or propoxur resistance, its RS genotype frequency was correlated with propoxur resistance (r^2^ = 0.815, P = 0.036). And the HWE test showed the A328S mutation is linked with V185M, also with G247S mutation. This suggested that these three mutations may contribute synergistically to propoxur resistance. The T682A mutation was negatively correlated with propoxur (r^2^ = 0.788, P = 0.045) resistance. Knowledge of these mutations may help design strategies for managing pesticide resistance in wild mosquito populations.

## Introduction

Acetylcholinesterase (AChE, EC 3.1.1.7) is a key enzyme in the nervous system of both vertebrates and invertebrates that terminates nerve impulses by catalyzing the hydrolysis of the neurotransmitter acetylcholine (ACh) released from the presynaptic membrane [1]. The inhibition of AChE by organophosphate and carbamate insecticides leads to the desensitization of the ACh receptor, thereby blocking nerve signal transmission. Organophosphates and carbamates have structures analogous to ACh and inhibit AChE competitively at the active site. Hydrolysis of these pesticide compounds retards the reactivation of the enzyme or inactivates it [2]. The extensive use of organophosphate and carbamate insecticides has resulted in the development of high levels of resistance to them among insects [3], [4], [5], [6].

Ace1 is the key AChE gene in insects. Several studies have found evidence that a point mutation in the ace1 gene is associated with resistance to organophosphate and carbamate pesticides. This point mutation changes the structure of AChE making it insensitive to these insecticides. The first report of this mutation conferring insecticide resistance was in the two-spotted spider mite in 1964 [7]. Subsequent studies have demonstrated that many insect species have developed resistance to organophosphate and carbamate pesticides through decreased sensitivity of AChE [8], including many mosquito species, such as *Anopheles gambiae* [9], *Cx. pipiens* [10], [11], *Cx. pipiens quinquefasciatus* [12], *Cx. tritaeniorhynchus* and *Cx. vishnui* [13]. However, so far, only three ace1 mutations, G119S, F331W and F290V (*T. californica* numbering) [13], [14], [15], [16], have been confirmed to be involved in such resistance in mosquito species. Determining the mutations that confer resistance to specific pesticides is important to designing effective strategies for managing pesticide resistance. *Cx. pipiens quinquefasciatus* is the main mosquito species in urban environments in southern China and one of the most studied in terms of insecticide resistance. We here report the results of an investigation of mutations in the ace1 gene in five wild Chinese populations of *Cx. pipiens quinquefasciatus*. Knowledge of these mutations may have practical benefits for reducing pesticide resistance in this species.

## Results

### Resistance of the Five Mosquito Populations to Dichlorvos and Propoxur

LC_50_ values of the five different populations ranged from 0.266 to 1.67 ppm for dichlorvos, and from 0.279 to 1.27 ppm for propoxur (Table 1). The HC strain had the lowest LC_50_ and was the most susceptible to both dichlorvos and propoxur. The SF strain had an LC_50_ to dichlorvos of 1.67 ppm and was 17.6 times more resistant to dichlorvos than the laboratory strain (LC_50_ 0.095 ppm). The GN strain had an LC_50_ to propoxur of 1.27 ppm and was 11.0 times more resistant to propoxur than the laboratory strain (LC_50_ 0.115 ppm). The HP strain was 7.89 times more resistant to dichlorvos, and 4.62 times more resistant to propoxur, than the laboratory strain. The QB strain was 13.1 times more resistant to dichlorvos, and 5.20 times more resistant to propoxur than the laboratory strain.

**Table 1:** Levels of dichlorvos and propoxur resistance in five populations of *Cx. pipiens quinquefasciatus.*

Population^1^	Insecticide	LC_50_ and LC_90_ (ppm) (95% CL)^2^	Regression Equation	Slope	Standard Deviation	χ^2^	P	RR^3^
LA	Dichlorvos	0.095^4^						1
	Propoxur	0.115^5^						1
GN	Dichlorvos	1.189 (0.923, 1.521) 3.376 (2.475, 5.505)	Y = −0.212+2.827x	2.827	0.200	58.08	<0.01	12.52
	Propoxur	1.266 (1.073, 1.595) 3.672 (2.576, 7.043)	Y = −0.284+2.772x	2.772	0.282	41.21	0.002	11.01
HP	Dichlorvos	0.750 (0.661, 0.853) 2.499 (2.067, 3.164)	Y = 0.306+2.453x	2.453	0.171	8.452	0.934	7.895
	Propoxur	0.531 (0.500, 0.564) 0.894 (0.820, 0.997)	Y = 1.557+5.668x	5.668	0.429	9.720	0.881	4.617
HC	Dichlorvos	0.266 (0.224, 0.309) 1.032 (0.835, 1.366)	Y = 1.252+2.175x	2.175	0.197	7.039	0.900	2.800
	Propoxur	0.279 (0.238, 0.320) 0.947 (0.755, 1.329)	Y = 1.338+2.413x	2.413	0.208	29.80	0.054	2.426
QB	Dichlorvos	1.240 (1.051, 1.464) 6.047 (4.609, 8.661)	Y = −0.174+1.862x	1.862	0.118	38.81	0.038	13.05
	Propoxur	0.598 (0.559, 0.639) 0.895 (0.813, 1.033)	Y = 1.635+7.319x	7.319	0.592	23.91	0.032	5.200
SF	Dichlorvos	1.672 (1.520, 1.822) 4.365 (3.905, 4.999)	Y = −0.687+3.076x	3.076	0.208	17.46	0.737	17.60
	Propoxur	0.785 (0.738, 0.837) 1.423 (1.278, 1.639)	Y = 0.522+4.959x	4.959	0.400	19.02	0.213	6.826

1 LA = Lab strain; GN = Guangzhou Nansha; HP = Haikou Poxiang; HC = Haikou Changliu; QB = Qionghai Boao; SF = Sanya Fenghuang.

2 CL = confidence limits.

3 RR = Resistance Ratio.

4 and ^5^are coming from Li Chunxiao’ dissertation [39].

### Identification of Ace1 Mutations

**Figure 1:**
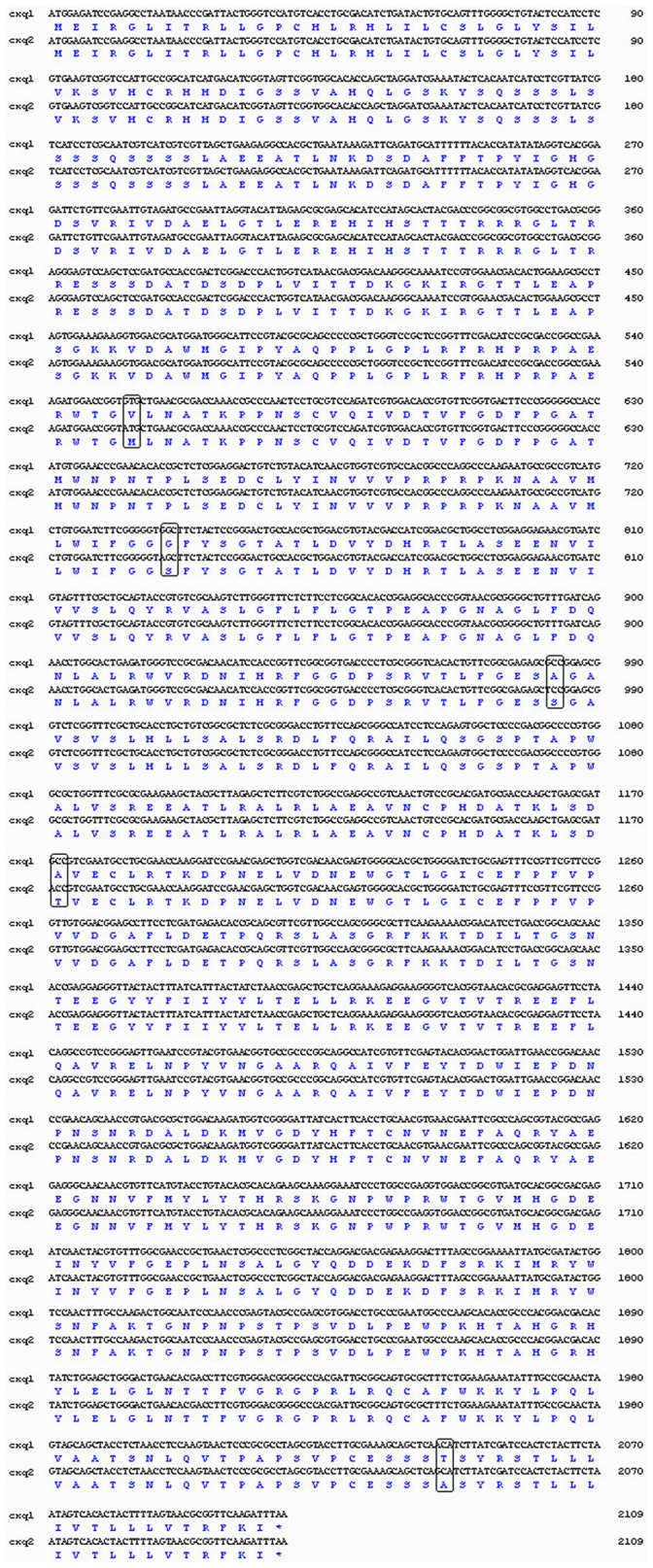
Alignment of nucleotide and amino acid sequences of *Cx. pipiens quinquefasciatus*. Cxq1 is the template nucleotide sequence (no amino acid mutation) and Cxq2 the mutant nucleotide sequence. Nucleotides are numbered on the first line, amino acids on the second. The five mutations are shown in the black frames.

To identify mutations in the ace1 gene, the cDNA of a pooled sample of mosquitoes from each of the five populations was cloned and sequenced. Five mutations (V185M, G247S, A328S, A391T, and T682A) in the pooled ace1 gene were identified (Figure 1), and the sequence was deposited in GenBank under the accession number KF680946. Note that this identification of 5 mutations does not imply all occur in the same ace1 gene. The V185M mutation was GTG to ATG, the G247S mutation was GGC to AGC, the A328S mutation was GCC to TCC, the A391T mutation was GCC to ACC, and the T682A mutation was ACA to GCA.

### Polymorphism of the Ace1 Gene in Natural Population

#### 1. Determination of the allele frequencies

The allele frequencies of each mutation were determined by specific PCR amplification using the primers Cx-ace2-F, Cx-ace2-R and Cx-ace3-F, Cx-ace3-R on the cDNA obtained from individual mosquitoes. Genotypes of each mosquito in each population was determined by sequencing, and mutation frequencies (R%) computed (Table 2). We can see from Table 2 that the V185M, A328S and T682A mutations were present at different frequencies in all five strains. However, the A391T mutation was only found in the HP and QB strains, and the G247S mutation was found in all but the HC strain.

**Table 2:** Mutation frequencies of five ace1 gene mutations and HWE test in five populations of *Cx. pipiens quinquefasciatus*.

Mutations	Strains	Numbers	Mutation frequency (R %)	P-value of HWE		HWE across strains	
				deficit	excess	χ^2^	P
V185M	GN	36	25.0	1.00	0.06	9.84	0.45
	HP	33	6.10	1.00	0.91		
	HC	30	16.7	0.15	0.99		
	QB	31	11.3	1.00	0.68		
	SF	30	23.3	0.50	0.84		
G247S	GN	36	18.1	0.73	0.70	0.00	1.00
	HP	33	1.50	No^1^	No		
	HC	30	0.00	No	No		
	QB	30	11.7	1.00	0.67		
	SF	30	5.00	1.00	0.95		
A328S	GN	36	47.2	0.83	0.39	1.77	1.00
	HP	33	19.7	0.77	0.66		
	HC	34	2.90	1.00	0.98		
	QB	30	11.7	1.00	0.67		
	SF	30	16.7	1.00	0.41		
A391T	GN	15	0.00	No	No	7.79	0.10
	HP	22	47.7	1.00	0.02		
	HC	13	0.00	No	No		
	QB	23	54.3	0.84	0.45		
	SF	22	0.00	No	No		
T682A	GN	35	18.6	0.01	1.00	18.0	0.06
	HP	32	51.6	0.73	0.53		
	HC	36	48.6	0.90	0.28		
	QB	33	39.4	0.03	1.00		
	SF	31	24.2	0.89	0.44		

1 No is no information, the reasons are because the site is homozygous for one mutation in this sample or because there is a single heterozygote.

#### 2. Hardy–Weinberg Equilibrium (HWE) test and genetic linkage analysis of the mutations

The results of GENEPOP software analysis of HWE and genetic linkage of the acetylcholinesterase gene mutations are shown in Tables 2 and 3. The HWE test indicates the QB and GN populations have a heterozygote deficit with respect to the T682A mutation (P<0.05), and the HP population a heterozygote excess with respect to the A391T mutation (P<0.05). Mutations in all other populations did not deviate from the HWE and none of the five mutations deviated from the HWE across all populations (P>0.05).

**Table 3:** P-value for linkage disequilibrium of each pair of loci across all populations (Fisher’s method).

Locus pair	χ^2^	df	P-Value
V185M & G247S	11.237	8	0.1887
V185M & A328S	23.804	10	0.0081
G247S & A328S	13.988	8	0.0821
V185M & A391T	7.5840	2	0.0226
G247S & A391T	3.4992	2	0.1738
A328S & A391T	5.8691	4	0.2091
V185M & T682A	4.8208	10	0.9028
G247S & T682A	2.4273	6	0.8765
A328S & T682A	14.160	10	0.1658
A391T & T682A	1.1946	4	0.8790

Results of linkage disequilibrium analysis of the five mutations are shown in Table 3. Evidence of linkage disequilibrium was found for V185M with respect to the A328S and A391T mutations (P<0.05), The G247S and A328S mutations’ linkage disequilibrium P-value was 0.0821, only slightly above 0.05. This suggests that these two mutations might exist in the same gene. Our sequencing data indicated that that these two mutations do indeed occur in the same ace1 gene in some mosquitoes. But the conclusion had to be confirmed by more data. All other gene polymorphism was randomly distributed.

### Correlation of Resistance with Mutation Frequencies

**Figure 2:**
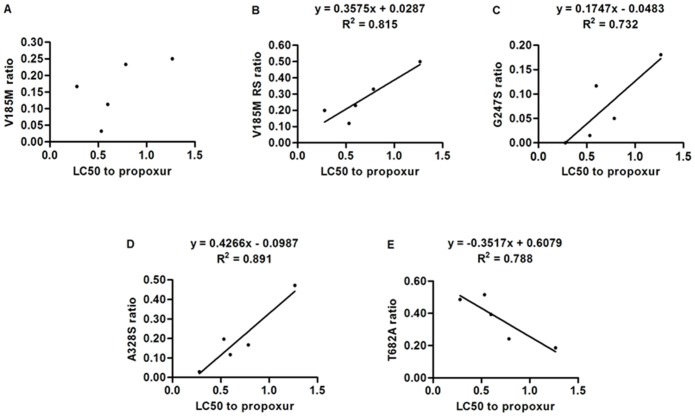
Linear regression of the relationship between resistance levels (LC50) and mutation ratios. Resistance levels to propoxur are plotted against the ratios of V185M (A), the RS ratio of V185M (B), G247S (C), A328S (D), and T682A (E).

The correlation between resistance to dichlorvos and propoxur and the frequencies of four mutations (V185M, G247S, A328S, T682A) are shown in Figure 2 and Table 4. The four mutations’ frequencies were all not significantly correlated with dichlorvos resistance. Although the frequency of the V185M mutation was uncorrelated with propoxur resistance (Figure 2 A), its RS genotype frequency was (r^2^ = 0.815, P = 0.036) (Figure 2 B). The correlation between the frequency of the G247S mutation and propoxur resistance was close to significance (r^2^ = 0.732, P = 0.065), and there was a significant linear relationship between the frequency of the A328S mutation and propoxur resistance (r^2^ = 0.891, P = 0.016) (Figure 2 C, D). The frequency of the T682A mutation was negatively correlated with propoxur (r^2^ = 0.788, P = 0.045) resistance (Figure 2 E).

**Table 4:** The analysis results of correlation between propoxur LC50 and mutation frequencies.

Mutations	Insecticide	R (95% CL^1^)	R^2^	P	Significance^2^
V185M	Propoxur	0.647(−0.549,0.974)	0.419	0.238	No
V185M (RS%)	Propoxur	0.903(0.101,0.994)	0.815	0.036	Yes
G247S	Propoxur	0.855(−0.110,0.990)	0.732	0.065	No
A328S	Propoxur	0.944(0.366,0.996)	0.891	0.016	Yes
T682A	Propoxur	−0.887(−0.993, −0.023)	0.788	0.045	Yes

1 CL = confidence limits.

2 α = 0.05.

### 3D Models of Mutations and Structural changes at the Catalytic Site

**Figure 3:**
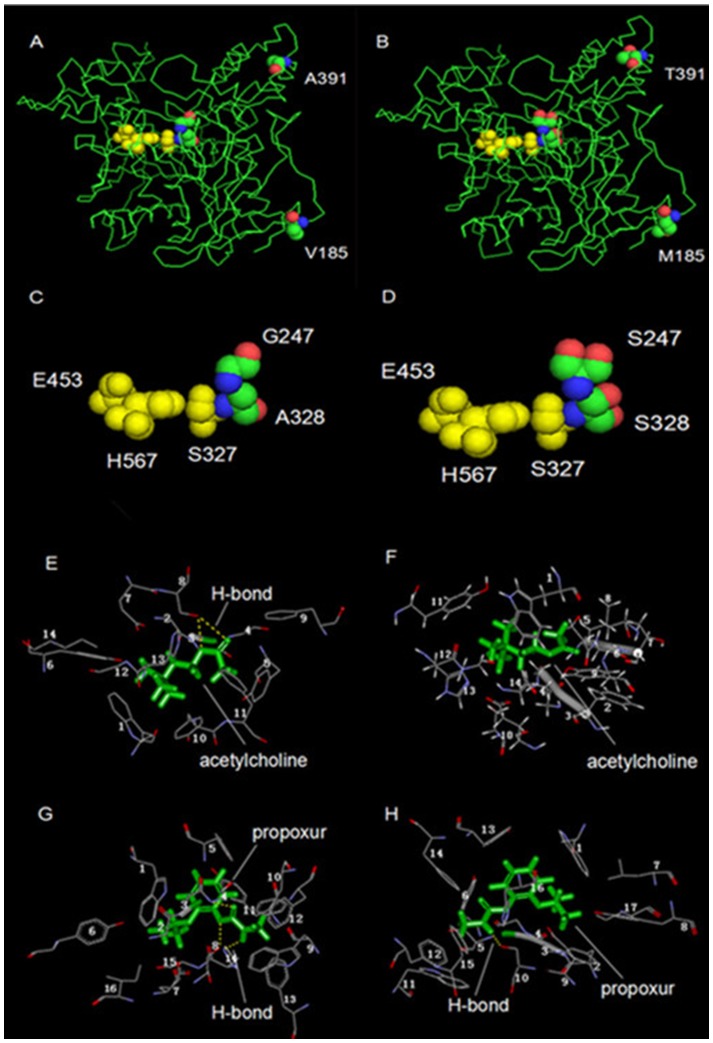
Three dimensional model of the AChE of *Cx. pipiens quinquefasciatus* based on the structure of *T. californica* (PDB: 3ZV7). The four mutations are shown as red, green and blue van der Waals spheres and the catalytic triad (S327, H567, E453; S200, H440, E327 in *T. californica*) is shown in yellow. A–D illustrates the four mutations. A shows the V185 and A391 positions and B the M185 and T391 mutations. C and D show the G247S and A328S mutations, and the catalytic triad. E–H shows changes in the enzyme–substrate complex; Ach (E, F) and propoxur (G, H) are shown in green and the H-bond as yellow dotted lines. Amino acids are marked with numbers. E1–14 (wild-type enzyme) are W212, G245, G246, G247, Y249, Y258, E326, S327, F416, Y456, F457, H567, G568, I571 respectively; The two H-bonds were composed of S327 Ogamma and O3, S327 Ogamma and O4. F1–14 (G247S/A328S mutant) are W212, F244, G245, G246, S250, G251, T252, L255, Y258, S327, Y456, H567, G568, I571 respectively; G1–16 (wild-type enzyme) are W212, G245, G246, G247, Y249, Y258, E326, S327, W360, F416, Y456, F457, F527, H567, G568, I571 respectively; The three H-bonds were composed of G247(4) NH and O13, S327(8) Ogamma and O11, H567(14) and NH27. H1–17 (G247S/A328S mutant) are W212, F244, G245, G246, S247, Y249, L255, Y258, E326, S327, W360, F416, Y456, F457, F527, H567, I571 respectively. The only H-bond was composed of S327(10) Ogamma and NH27.

A 3D model was made of the *Cx. pipiens quinquefasciatus* ace1 gene sequence allowing the location and structure of four mutations to be visualized (Figure 3). The V185M and A391T mutations are distant from the active site of the enzyme-catalytic triad (S327, H567, E453; S200, H440, E327 in *T. californica*) (Figure 3A, B). The other two mutations, G247S and A328S, are close to the catalytic site (Figure 3C, D) and could therefore potentially affect the binding between AChE and its substrates (Ach: ZINC3079336 and propoxur: ZINC1590885). Figure 3E-H illustrates the change in amino acids and H-bonds associated with the G247S and A328S mutations. These two substitutions change the amino acids present at catalytic sites removing the two H-bonds (S327(8)O_γ_-O3, S327(8)O_γ_-O4) between AChE and Ach (Figure 3E, F) and reducing the three H-bonds between AChE and propoxur (G247(4)-O13, S327(8)O_γ_-O11, H567(14)-NH27) to one (S327(10)O_γ_-NH27) (Figure 3G, H). Hence, these two mutations could have a major effect on the catalytic activity of the AChE enzyme.

## Discussion

The indiscriminate use of insecticides over more than half a century has resulted in high levels of insecticide resistance in many mosquito species [13], [17], [18]. We tested the resistance of five Chinese *Cx. pipiens quinquefasciatus* populations to dichlorvos and propoxur. Our results show that, compared to a laboratory strain, these five populations displayed a 2.80- to 17.6-fold resistance to dichlorvos and 2.43- to 11.0-fold resistance to propoxur. The frequent use of these insecticides has created an intense selection pressure for traits that confer resistance to them, such as changes in behavior, epidermal structure, metabolic enzymes and target site mutations. Resistance may be conferred by any one, or more than one of these mechanisms. Osta et al (2012) found that the dramatic reduction in the frequency of the G119S (*T. californica* numbering) mutation in *Culex pipiens* mosquitoes was probably due to the increased use of pyrethroids over organosphosphate insecticides [19]. Therefore, alternating between different kinds of insecticides is one way of minimizing the development of resistance to any one kind.

We used cloning and sequencing to identify five point mutations in the ace1 gene of Chinese *Cx. pipiens quinquefasciatus*. HWE tests suggest that these five mutations do not deviate from the HWE across all populations. However, the tests also indicated that the QB and GN populations were deficient in heterozygotes with respect to the T682A mutation and that HP population had an excess of heterozygotes with respect to the A391T mutation (P<0.05). Further work will be required to determine the reasons for these departures from the HWE. Linkage disequilibrium analysis indicated significant linkage between the V185M mutation and the A328S and A391T mutations. Although linkage between other mutations was statistically insignificant, that between the G247S and A328S mutations was nearly so (P = 0.0821). Our sequencing results suggest that these two mutations occur within the same ace1 gene in some mosquitoes but further work is required to confirm this hypothesis.

These results are the first report of the V185M mutation in *Cx. pipiens quinquefasciatus*. Although there was no apparent correlation between the frequency of this mutation and resistance to dichlorvos and propoxur, the frequency of its RS genotype was significantly correlated with propoxur resistance (r^2^ = 0.815, P = 0.036). Although the 3D model indicates that V185M is located far from the active site, the positive linear relationship between its RS genotype frequency and propoxur resistance, and its apparent linkage with the A328S mutation suggest that it may be involved in propoxur resistance. Of course, we cannot rule out the possibility that insecticide resistance involves multiple duplication of the ace1 gene. Further research needs be required to determine how this might affect the catalytic center.

Our results (Figure 2, Table 4) suggest that the G247S mutation is not associated with propoxur resistance and that the A328S mutation is. The G247S mutation corresponds to G119S in *T. californica* which has been associated with insecticide resistance in mosquitoes by several authors [20], [21]. The G119 position is part of the oxyanion hole (G118, G119, and A201 in *T. californica*), close to the catalytic Serine (S200) where a G to S substitution would reduce accessibility to inhibitors and substrate by steric hindrance. S119 is close enough to the catalytic residues to alter the presentation of inhibitors and substrates. This could be the reason this mutation confers resistance to some insecticides [22], [23]. Although the correlation between the frequency of the G247S mutation and propoxur resistance was not statistically significant (P = 0.065), numerous prior publications have reported such an association [9], [10], [12] and noted that this mutation is often combined with other mutations in resistant strains. Therefore, we suspect that G247S probably is involved in propoxur resistance. We may have failed to detect a significant correlation between the frequency of this mutation and resistance because of its low frequency in our sample, which could be because most mosquitoes carrying it were heterozygotes. Furthermore, the resistance conferred by this mutation may be nearly recessive under certain bioassay conditions [24].

The A328 position corresponds to the A201 position in *T. californica*, which is located within the active gorge of the enzyme, close to the catalytic site,and is a part of the oxyanion hole. Li et al (2009) also found the A328S mutation in *Cx. pipiens pallens* and made a three-dimensional model of AChE to visualize this mutation. However, they did not demonstrate a relationship between the A328S mutation and resistance [25]. Khajehali et al (2010) found the corresponding A201S mutation in *Tetranychus urticae* Koch, and demonstrated that this was possibly involved in resistance to organophosphorus and carbamate insecticides [26]. Our results suggest that this mutation is involved in propoxur resistance (r^2^ = 0.891, P = 0.016). The linkage disequilibrium and sequencing results indicate that A328S and G247S mutations exist in a same ace1 gene in some mosquitoes, which suggests that they may work synergistically. The G119 and A201 positions (*T. californica* numbering) are both part of the oxyanion hole, and could therefore both contribute an amide nitrogen to form bonds that could stabilize the enzyme–substrate complex. The substitution of serine for glycine and alanine may change the conjunctions, conferring resistance to some insecticides [23], [27]. We can see from Figure 3 that these substitutions could decrease the numbers of H-bonds between enzyme and substrate. H-bonds are the strongest force between molecules so a reduction in these could reduce enzyme-substrate stability and interfere with the catalytic reaction.

The G119S mutation was the first mutation found in mosquito vectors [10]. Previous studies indicated that this mutation would incur a high fitness cost [28], however, although the cost of resistance is often high at the beginning of selection when resistance is unstable, the cost reduces and resistance stabilizes with increasing duration of exposure to insecticides [29]. Other mutations can play an important role in this process. Mutero et al (1994) found that high levels of resistance were obtained by the combination of several point mutations [1] and Menozzi et al (2004) demonstrated that combining mutations could increase insecticide resistance in *Drosophila melanogaster* [30]. Our results show that the A328S mutation (A201S in *T. californica*) may work synergistically with the G119S mutation in the oxyanion hole. It’s possible that the A328S mutation compensates for some of the fitness costs incurred by the G119S mutation. This is a fascinating question but further *in vitro* assays are required to confirm this hypothesis.

The A391T mutation was found only in the HP and QB populations, which had moderate LC_50_ values and in which it had a frequency of around 0.500. The genetic linkage analysis indicates a linkage between this mutation and V185M, however, in view of the small sample size further work is required to confirm this. The three-dimensional model revealed that the A391 mutation is distant from the active site. This indicates that this mutation is unlikely to affect catalytic activity and is probably not involved in dichlorvos and propoxur resistance. How this mutation developed and its function, if any, in pesticide resistance requires further investigation.

Our results provide the first evidence of the T682A mutation in *Cx. pipiens quinquefasciatus*. The frequency of this mutation was negatively correlated with propoxur resistance (r^2^ = 0.788, P = 0.045). Fournier et al (1988) found that AChE in *Drosophila melanogaster* was composed of two, non-covalently associated, polypeptides of 55 and 16 kDa. AChE is an amphiphilic protein linked to the membrane of neuronal cholinergic synapses via a glycolipid anchor at the C-terminal end of the 55 kDa polypeptide [31]. Nabeshima et al (2004) found an I697M replacement near the C-terminus (Ile701) in *Culex tritaeniorhynchus*, but considered that this was unlikely to be the cause of AChE insensitivity [32]. Our results also indicate that the T682A mutation is near the C-terminus of AChE, and that the frequency of this mutation is negatively correlated with propoxur resistance. Despite its negative correlation with resistance, it’s possible that this mutation may change the C-terminus structure of AChE thereby reducing its attachment to the membrane and the stability of enzyme. We don’t know whether this mutation works in combination with the other four mutations or not, or if its apparent negative relationship with resistance is related to fitness costs.

In conclusion, we found five ace1 gene mutations in *Cx. pipiens quinquefasciatus* that are correlated with propoxur, but not dichlorvos resistance. The V185M mutation was first confirmed in *Cx. pipiens quinquefasciatus* and may be involed in propoxur resistance. The allele frequencies of the G247S and A328S mutations were positively correlated with resistance. So the G247S and A328S mutations are also likely to confer propoxur resistance. The A391T mutation appears unrelated to dichlorvos and propoxur resistance and the T682A mutation appears negatively correlated with resistance to propoxur. Identifying the mutations that confer resistance to specific insecticides can inform the choice of insecticides for a given insect population, thereby reducing the development of resistance and improving the efficacy of control.

## Materials and Methods

### Statement of Ethical Approval

No ethical approval was required as no regulated animals were used in this study. Pre-permission (May–September 2012) was granted for observation, collection and field research on mosquitoes in Guangdong and Hainan Provinces, which was conducted as part of the Infective Diseases Prevention and Cure Project of the China National Ministry of Public Health (No.2008ZX10004 and No.2012ZX10004219). All field studies on *Cx. pipiens quinquefasciatus* were authorized by Guangdong and Hainan Provincial CDC Committees for Animal Welfare and Animal Ethics (address: 176 Xingang West Road, Guangzhou, Guangdong province, and 44 Haifu Road, Haikou, Hainan province, P. R. China).

### Mosquito Strains

Specimens of *Cx. pipiens quinquefasciatus* were collected from five different field sites; Guangzhou Nansha (E113°29′29.35″, N22°48′4.13″) and Haikou Poxiang (E110°19′33.79″, N19°59′55.07″) in May 2012, and Haikou Changliu (E110°11′50.36″, N20°0′50.25″), Qionghai Boao (E110°34′57.13″, N19°09′42.07″) and Sanya Fenghuang (E109°26′54.38″, N18°18′2.91″) in September 2012. The susceptible strain had been reared in an insectarium for more than 10 years without exposure to any insecticides.

*Cx. pipiens quinquefasciatus* larvae were collected at each field site and reared to adulthood. Some wild caught female adults were frozen in liquid nitrogen for subsequent testing.

### Bioassay

Bioassays were conducted by putting thirty late 3^rd^ or early 4^th^ instar larvae into pans containing 200 ml water. Measured quantities of insecticides were added to each pan using an automatic pipette according to the methods specified by the WHO [33]. Larval mortality was recorded 24 h after each treatment. No food was offered to larvae during bioassays. Larvae were maintained in the laboratory under a 14L:10D photoperiod, 75% relative humidity and temperature of 26±1°C during bioassays. Bioassays of each insecticide were repeated three times. Statistical analyses were performed using SPSS software version 13.

### Extraction of RNA and cDNA Synthesis

Total RNA was extracted from specimens from each population with Trizol reagent (GBT) following the manufacturer’s protocol and cDNA synthesized from the total RNA with cDNA synthesis kit (TaKaRa). The cDNA was stored at −20°C.

### PCR Amplification

**Figure 4:**
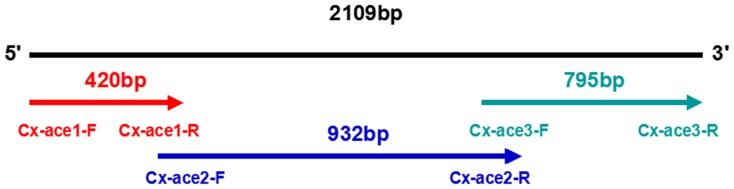
Schematic diagram of the amplification of the ace1 gene. The complete sequence was 2109(black), and the three sections are indicated by red, blue and green arrows.

Gene specific primers based on the published insecticide resistant sequence of the *Cx. pipiens quinquefasciatus* ace1 gene (GenBank Accession No.:CQ753634.1, this includes a G119S mutation related to propoxur resistance) were designed in NCBI-Primer-BLAST and used to amplify the ace1 gene of each population. The ace1 gene is 2109 bp and is divided into three sections (Figure 4). The primers used are shown in Table 5.

**Table 5:** The primers used to amplify the *Cx. pipiens quinquefasciatus* ace1 gene.

Primers	5′→3′Sequence	length (bp)	PCR parameters
Cx-ace1-F	ATGGAGATCCGAGGCCTAAT	420	94°C,5 min; 94°C,30 s; 62°C,30 s; 72°C,1 min,35 cycles; 72°C,7 min.
Cx-ace1-R	GCCCTTGTCCGTCGTTATG		
Cx-ace2-F	CGGACCCACTGGTCATAACG	932	94°C,5 min; 94°C,30 s; 65°C,30 s; 72°C,1 min,35 cycles; 72°C,7 min.
Cx-ace2-R	ACCCTCCTCGGTGTTGCTG		
Cx-ace3-F	CGCTTCAAGAAAACGGA	795	94°C,5 min; 94°C,30 s; 55°C,30 s; 72°C,1 min,35 cycles; 72°C,7 min.
Cx-ace3-R	TTAAATCTTGAACCGCGT		

### Cloning and Sequencing of PCR Products

PCR products were purified using a universal DNA purification kit (TIANGEN) and the purified products were ligated into the pEASY-T1 vector (TRANSGEN). The recombinant plasmids were cloned into Trans1-T1 competent cells (TRANSGEN). The microbials were spread on LB solid medium (including ampicillin, X-gal, IPTG) and cultured overnight. White clones were selected, placed in LB liquid medium and cultured to turbidity. Positive clones were identified by PCR using M13 forward and reverse primers and sequenced by Sangon Biotech [25]. Based on the discovery of clones, the genotype of individual mosquitoes was determined for each amino acid position by specific PCR amplification and sequencing. In this procedure, a single mosquito’s RNA was extracted and reversed transcribed to cDNA, then amplified by specific PCR before sequencing. Calculated mutation frequencies were based on the sequencing results.

### Hardy–Weinberg Equilibrium (HWE) Test and Genetic Linkage Analysis of the Mutations

The Hardy–Weinberg equilibrium (HWE) describes the theoretical frequency of two alleles of a single locus in the absence of mutation and selection after one generation of random mating in an indefinitely large population with discrete generations [34]. We used GENEPOP software to analyze the HWE and genetic linkage of mutations.

### Correlation of Pesticide Resistance with the Allele Frequency of Different Mutations in the Five Mosquito Populations

The resistance (LC_50_) of the five populations to propoxur and dichlorvos was determined by bioassay and the allele frequencies of the various mutations were determined by gene specific amplification and sequencing as described above. The LC_50_ of a laboratory strain that had not been exposed to either pesticide was also determined to serve as a control. Correlations between resistance and mutation frequency were analyzed using Graphpad Prism 5.

### Three-dimensional (3D) Modeling

The ace1 gene sequence of *Cx. pipiens quinquefasciatus* was translated into an amino acid sequence of AChE1. The protein was then modeled against the 3D structure of *T. californica* AChE (PDB accession no. 3ZV7) using the SWISS-MODEL homology modeling server (http://swissmodel.expasy.org/) [35], [36], [37] and molecular docking using the LibDock utility in Discovery Studio 2.5 [38].
